# Interjoint Coordination at Different Squatting Speeds in Healthy Adults

**DOI:** 10.7759/cureus.67620

**Published:** 2024-08-23

**Authors:** Akihiko Kirishima, Masaya Anan

**Affiliations:** 1 Department of Rehabilitation Medicine, Nishi-Hiroshima Rehabilitation Hospital, Hiroshima, JPN; 2 Faculty of Welfare and Health Science, Oita University, Oita, JPN

**Keywords:** interjoint coordination, speed, joint moment, continuous relative phase, squatting

## Abstract

Background

Squatting is commonly used in various settings to enhance muscle strength and performance. Both fast and slow squats have advantages as training to improve muscle function in the lower extremity muscles. Movement speed affects the variability of interjoint coordination and decreased variability can lead to overuse injuries owing to repetitive mechanical loading on the lower extremity joints. However, only a few studies have focused on interjoint coordination during squatting. This study aimed to clarify the kinematic and kinetic differences, as well as the interjoint coordination, during squatting at different speeds.

Methodology

Healthy young participants with no locomotor disease were recruited to perform descending parallel squats at different speeds (one, three, and five seconds) using a 3D motion analysis system and force plates. Joint moments and continuous relative phases were calculated and compared between the conditions.

Results

There were no significant differences in the mean values of lower limb joint moments among the three speed conditions. However, the mean absolute values of the continuous relative phase between the ankle and hip joints and the mean standard deviation of the continuous relative phase between each lower limb joint were significantly lower in the high-speed condition than in the medium- and low-speed conditions. Additionally, in the high-speed condition, the knee joint moved ahead of the hip joint in the knee-hip joint phase coordination pattern.

Conclusions

The joint load per unit time remained constant across all speed conditions. High-speed squatting may adapt to mechanical loading on the joints, although the knee joint moves ahead of the hip joint, exhibiting a highly coordinated movement. Conversely, low-speed squatting may reduce the risk of disability owing to the high variability of interjoint coordination. Therefore, squatting training should be based on individual characteristics and objectives.

## Introduction

Squatting is a fundamental motion in sports and activities of daily living (ADLs). It is used in a wide range of situations as a form of training to increase muscle strength and functional ability [1]. Squatting involves the contraction of the hip extensors, abductors, adductors, quadriceps, and triceps surae muscles [2], making it effective for training in clinical settings because most ADLs require lower-limb muscle coordination [3,4].

When performed correctly, squatting is a safe movement because the compressive, shear, and rotational forces acting on the joints can be modulated by adjusting barbell positioning, load, squat depth, stance width, direction of gaze, fatigue, and movement speed [5]. Specifically, high-speed squat training increases hip and ankle joint power in vertical jumping tasks, as well as peak knee joint velocity and total body power in long jumping tasks [6]. Conversely, low-speed squat training increases knee joint torque and quadriceps muscle thickness during vertical jumping tasks and enhances hip joint moment and one-repetition maximum weights during the squatting task [6,7]. These findings indicate that both high-speed and low-speed squatting have advantages in terms of training.

However, squatting poses a risk of injury. These include the avulsion of the reflected head of the rectus femoris muscle and joint lip injury in the hip joint [8]. Injuries to the knee joint can include rupture of the quadriceps tendon [9]. Additionally, the faster the squatting, the greater the anteroposterior shear and compressive forces on the knee [10,11]. Fast and uncontrolled squatting can generate excessive strain and shear forces on the cruciate and collateral ligaments, potentially damaging them [12].

ADLs require lower-limb muscle coordination, which is crucial for understanding the mechanical load on the joints and the associated risk of injury. For instance, in walking tasks, interjoint coordination between the trunk and pelvis and between the lower extremities is affected by walking speed, showing more in-phase coordination at low speeds and out-of-phase coordination at high speeds [13,14]. High interjoint coordination contributes to shock absorption during the early phase of stance [15]. Conversely, reduced variability in interjoint coordination can cause excessive repetitive mechanical loads on the joints, leading to the overuse of disorders [16]. Few studies have focused on interjoint coordination at different squatting speeds. Squatting involves coordinated interjoint movement of the lower extremity joints, including the hip, knee, and ankle joints. Different squatting speeds may alter interjoint coordination, potentially increasing the risk of injury. Interjoint coordination can be characterized by the continuous relative phase (CRP), a measure commonly used for clinical purposes [17,18]. The CRP evaluates interjoint coordination by quantifying the trajectory of the phase plane and includes both position and velocity signals, providing spatiotemporal information [19]. The normalized joint angles and joint angular velocities at each time point over the entire motion cycle were plotted in the phase plane, and the phase angle of one joint was subtracted from that of the other joint to obtain the CRP angle. The CRP angle indicates the relative in-phase and anti-phase between the two joints [16,20]. The variability of CRP can quantify the variability of interjoint coordination [16,21].

Therefore, this study was conducted on healthy young adults to clarify the kinematic and kinetic differences and interjoint coordination of the lower limbs during different squatting speeds. We hypothesized that high-speed squatting would make it difficult to control multiple joints, resulting in less interjoint coordination variability. Understanding the load and coordination structure of the lower limb joints may assist in the instruction of squat training to reduce the risk of injury.

## Materials and methods

Participants

Participants were 13 healthy young adults (mean age: 21.1 ± 0.47 years, height: 1.57 ± 0.07 m, mass: 56.8 ± 8.83 kg) with no locomotor diseases affecting task performance. The minimum sample size was estimated using G*Power3 software with an alpha value of 0.05 and a beta value of 0.20. Sample size calculations were based on an effect size of 0.8, as reported by Cohen [22], and the inclusion of a minimum of 12 participants was considered sufficient to detect significant differences between conditions. Exclusion criteria included trauma to the lower extremities or pain during task motion. In accordance with the regulations of the Ethics Committee of the Faculty of Welfare and Health Science at Oita University (approval number: F210045), each participant was informed of the purpose, content, and safety of the study before the experiment was conducted. Consent for participation in the experiment was obtained from all participants.

Apparatus and experimental procedure

This was a cross-sectional study and participants completed a single data collection session in a biomechanics laboratory. The participants performed parallel squats on two force plates at the depth where the thighs were parallel to the floor. Five trials in each condition were performed randomly at the following speed conditions on a metronome of 60 beats/minute: foot width between the anterior superior iliac spines, upper limbs folded in front of the chest, looking forward at all times, and not lifting the toes or heels off the floor. The duration of the three conditions was set as follows: one, three, and five seconds for the high-speed, medium-speed, and low-speed conditions, respectively. The task was performed while monitoring the movements in real time. Any deviation from the standard was considered a failure, and measurements were recorded until five successful trials were obtained for each condition.

Kinematic data were acquired at a sampling rate of 100 Hz using a 3D motion analysis system with 10 infrared cameras and reflection markers (Vicon, Oxford, UK). Kinetic data were acquired using two force plates (AMTI, Watertown, MA, USA) at a sampling rate of 1,000 Hz. A total of 46 infrared reflective markers were affixed to the anatomical landmarks throughout the body.

Data processing

The data were low-pass filtered using a Butterworth filter at 6 Hz for marker coordinates and 20 Hz for floor reaction force. The X-axis (right: +), Y-axis (anterior: +), and Z-axis (vertical: +) were used for the mediolateral, anteroposterior, and vertical directions, respectively. Data analysis software Vicon Nexus (Vicon, Oxford, UK) was used to calculate each parameter. From the marker coordinates obtained, a nine-rigid body link model was created for the head, thorax, pelvis, both thighs, both shanks, and both feet. The center of mass (COM) coordinates were calculated based on the inertia properties of the body segments [23]. The local coordinate system of the nine rigid bodies also defined the X-, Y-, and Z-axes in the same direction as the absolute coordinate system, and the foot, shank, thigh, pelvis, thorax, and head segments were defined.

The descending phase of squatting was analyzed. The descending phase was initiated when the vertical COM velocity fell below -5 mm/s and terminated when the vertical COM velocity exceeded -5 mm/s. The descending phase was normalized to 100%, and the joint angles, angular velocities, and moments of the hip, knee, and ankle joints were calculated. Joint moments are expressed as external moments, normalized by body mass and height, and averaged over the descending phase.

CRP was used as an index of spatiotemporal interjoint coordination [19]. A positive CRP value indicates that the distal joint is moving first, while a negative value indicates that the proximal joint is moving first [15,21]. When the mean absolute value of the ensemble CRP curve value (MARP) is close to 0°, the two joints are in-phase, and when it is close to 180°, the two joints are in antiphase [15,21]. The mean value of the standard deviation of the ensemble CRP curve values (DP) indicates the degree of variability [15,21].

$MARP=\frac{\sum_{i=1}^{p}\left |\phi _{REL.PHASE}\right |_{i}}{p}$

$DP=\frac{\sum_{i=1}^{p}SD_{i}}{p}$

Where p is the number of points in designated periods. Φ REL.PHASE is the CRP angle.

Statistical analysis

Statistical analyses were performed using the statistical software SPSS Statistics version 23 (IBM Japan, Tokyo, Japan). The Shapiro-Wilk test was used to check for the normality of the calculated parameters. If normality was found, a one-way analysis of variance for repeated measures was performed. If normality was not found, the Friedman test was performed. The Bonferroni method was used for multiple comparisons. The significance level was set at 0.05.

## Results

The mean values of the COM velocity increased significantly across low-, medium-, and high-speed conditions (low- and medium-speed conditions: p = 0.03, low- and high-speed conditions: p < 0.01, medium- and high-speed conditions: p = 0.03, F = 177.27, Table 1). The mean values of angles and moments of the hip, knee, and ankle joints did not show significant differences between the conditions (Table 1).

**Table 1:** Mean and SD of the COM velocity, joint angles, and joint moments. Mean ± SD; ^*^: p < 0.05: significant difference from the medium-speed condition; ^†^: p < 0.05: significant difference from the low-speed condition. COM = center of mass; SD = standard deviation

	High speed	Medium speed	Low speed	
COM velocity (m/s)	3.06 ± 1.76^*†^	1.37 ± 0.52^†^	0.84 ± 0.29^*^	F (2, 24) = 177.27
Hip joint angle (degrees)	67.92 ± 27.17	68.72 ± 24.71	70.16 ± 23.34	
Knee joint angle (degrees)	73.66 ± 31.38	75.29 ± 28.62	77.63 ± 26.72	
Ankle joint angle (degrees)	24.99 ± 9.04	25.61 ± 8.15	26.52 ± 7.45	
Hip joint flexion moment (Nm/kg m)	0.28 ± 0.23	0.27 ± 0.18	0.28 ± 0.17	
Knee joint flexion moment (Nm/kg m)	0.47 ± 0.19	0.47 ± 0.15	0.48 ± 0.13	
Ankle joint dorsiflexion moment (Nm/kg m)	0.07 ± 0.02	0.06 ± 0.02	0.06 ± 0.02	

**Figure 1:**
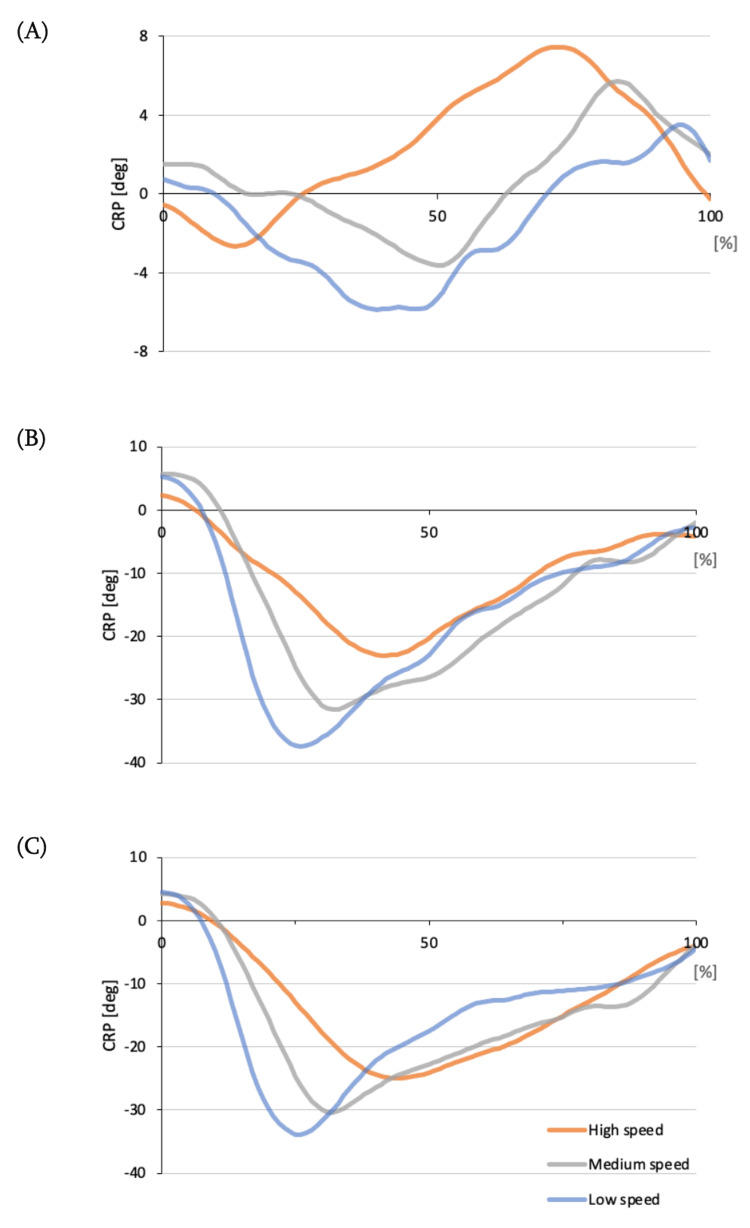
Interjoint coordination patterns. (A) The CRP between the knee-hip joints. (B) The CRP between the ankle-hip joints. (C) The CRP between the ankle-knee joints. Positive CRP values indicate that the distal joint is moving first, while negative values indicate that the proximal joint is moving first. CRP = continuous relative phase

A coordination pattern was observed between the knee and hip joints. Specifically, the knee joint preceded the hip joint in the high-speed condition, whereas the hip joint preceded the knee joint in the medium- and low-speed conditions (Figure 1). In all three conditions, the hip joint preceded the ankle joint, and the knee joint preceded the ankle joint. The MARP showed significantly lower values for the high-speed condition between the ankle and hip joints compared to the medium- (p = 0.03) and low-speed (p = 0.02) conditions (F = 0.43) (Table 2). The DP showed significantly lower values between the knee and hip joints for the high-speed condition compared to the medium- (p = 0.01) and low-speed (p < 0.01) conditions (F = 16.96); between the ankle and hip joints for the high-speed condition compared to the medium- (p = 0.02) and low-speed (p < 0.01) conditions (F = 15.06), and between the ankle and knee joints for the high-speed condition compared to the medium- (p = 0.02) and low-speed (p < 0.01) conditions (F = 6.01) (Table 2).

**Table 2:** Mean and SD of the MARP and DP. Mean ± SD; ^*^: p < 0.05: significant difference from the medium-speed condition; ^†^: p < 0.05: significant difference from the low-speed condition. DP = deviation phase; MARP = mean absolute relative phase; SD = standard deviation

	High speed	Medium speed	Low speed	
Knee-hip joint MARP (degrees)	7.26 ± 2.93	9.41 ± 3.88	9.71 ± 5.59	
Ankle-hip joint MARP (degrees)	15.43 ± 8.38^*†^	20.46 ± 11.43	21.63 ± 13.76	F (2, 24) = 0.43
Ankle-knee joint MARP (degrees)	14.64 ± 7.76	17.12 ± 8.10	16.42 ± 9.13	
Knee-hip joint DP (degrees)	3.52 ± 1.19^*†^	5.96 ± 1.97	6.50 ± 2.11	F (2, 24) = 16.96
Ankle-hip joint DP (degrees)	5.81 ± 1.73^*†^	9.12 ± 2.60	10.34 ± 3.94	F (2, 24) = 15.06
Ankle-knee joint DP (degrees)	3.90 ± 1.11^†^	5.85 ± 1.38	6.55 ± 2.73	F (2, 24) = 6.01

## Discussion

In this study, we clarified the kinematic and kinetic differences and the coordination between the lower limb joints during squatting under different velocity conditions in young healthy participants. The results showed that there were no significant differences in the mean values of joint moments, but there were significant differences in CRP levels between the conditions.

The mean COM velocity increased significantly in the low-, medium-, and high-speed conditions, and the mean lower extremity joint angles did not show significant differences between the conditions, indicating that the study was performed as prescribed. There were no significant differences in mean lower extremity joint moments between conditions, and lower extremity joint moments per unit time during the descending squatting phase remained constant, regardless of the speed condition. This suggests that the cumulative load increases with squatting at low speeds and that lower speeds are more effective in squat training. However, it has been reported that cumulative moment load on the hip joint on a daily basis is related to the progression of hip osteoarthritis [24], suggesting that squat training at low speeds with an awareness of cumulative load may lead to overload and increased risk of injury. In addition, knee joint moments were higher in all conditions compared to the hip joint. It has been reported that squatting is a knee joint extensor dominant exercise compared to other lower extremity exercises [25]. These suggest that actual squat training may increase the load on the knee joint more.

Regarding interjoint coordination, proximal joint priority movements were observed in the ankle-hip and ankle-knee joints under all conditions, as well as in the knee-hip joint medium- and low-speed conditions. This result was similar to that of previous studies that investigated interjoint coordination in the jumping squatting task [18]. On the other hand, knee joint, meaning distal joint, priority movements were observed in the knee-hip joint high-speed condition. It has been reported that the centrifugal contractile force of the knee extensor muscle group is greater than that of the hip extensor muscle group during the descending phase of squatting [26]. In addition, it has been reported that muscle tension must be reduced to increase the speed of movement based on the characteristics of force and speed [27]. These findings suggest that during squatting at high speed because there is no need to break the speed against gravity, the demand for muscle tension on the knee extensor muscle group, which is predominantly active during the descending phase, is smaller, and the knee flexion movement is preceded by the knee joint flexion movement.

The MARP was significantly lower and in-phase between the ankle-hip joints in the high-speed condition than in the medium- and low-speed conditions. This means that the independent movements of the two joints were reduced and the movements were highly interlocked. In a running task with obstacles of different heights, it has been reported that the magnitude of impact at landing is proportional to the magnitude of interlocking between joints and that high interlocking contributes to shock absorption [15]. Squatting at high speeds generates excessive shear, strain, and compressive forces that can damage the hip and knee joint structures [10-12], and it is possible that a highly interlocked movement pattern is generated to adapt to these loads on the joints.

The DP was significantly lower in the high-speed condition than in the medium- and low-speed conditions between each lower limb joint. Decreased variability means a decrease in the degree of freedom of movement due to mutual coordination between joints, which may cause injury when a critical threshold is reached owing to aging or other factors [28]. The low degree of freedom of coordination owing to decreased variability may lead to a concentration of compressive stress, which may cause overuse disorders. For example, patellofemoral joint pain and low back pain in running tasks [21,26]. In contrast, increased variability provides flexibility to accomplish task movements while coordinating coordination patterns [16,29] and is characterized as a functional form of neuromuscular flexibility to adapt to external perturbations in high-level athletes [18,30]. The presence of variability distributes compressive stresses applied to joints or tissues, thereby minimizing overuse injuries [16]. Based on these findings, we believe that squatting at medium and low speeds results in high variability in posture control over a fixed period. In contrast, squatting at repetitive high speeds reduced the variability of interjoint coordination, suggesting that the repetitive mechanical load on the joints may be greater, increasing the risk of injury. However, excessive variability may indicate structural instability of the joints; a healthy state is one in which the variability is within an appropriate range [16].

This study has two notable strengths. First, it employs the use of CRP in the analysis of squatting movements. Second, it examines interjoint coordination at different speeds among young healthy individuals. In this study, the joint moments per unit of time did not change, and squatting at high and low speeds exhibited different coordination patterns. These findings suggest that normal individuals may functionally change their interjoint coordination depending on speed conditions.

The limitations of this study include the lack of a detailed relationship between training and disability because barbell loading was not used and there was no clinical definition of the criteria for joint overload or overuse. In addition, because only healthy individuals were included in the study, changes in interjoint coordination with and without disabilities have not been clarified. Therefore, further clinical studies are warranted.

## Conclusions

The results revealed that the joint moments per unit of time did not change, and squatting at high and low speeds exhibited different coordination patterns. These findings suggest that normal subjects may functionally change their interjoint coordination depending on speed conditions. In addition, it is necessary to select a descent speed that is appropriate for the individual’s characteristics and purpose to provide movement guidance that reduces the risk of injury during squat training.
